# Effects of Acute Fructose Loading on Markers of Inflammation—A Pilot Study

**DOI:** 10.3390/nu13093110

**Published:** 2021-09-04

**Authors:** Camilla Olofsson, Monica Eriksson, Ann-Christin Bragfors Helin, Björn Anderstam, Nicola Orsini, Peter Stenvinkel, Neda Rajamand Ekberg

**Affiliations:** 1 Department of Molecular Medicine and Surgery, Karolinska Institutet, 171 77 Stockholm, Sweden; neda.ekberg@ki.se; 2 Division of Renal Medicine M99, CLINTEC, Karolinska Institutet, 171 77 Stockholm, Sweden; monica.i.eriksson@gmail.com (M.E.); annki.bh@me.com (A.-C.B.H.); bjorn.anderstam@icloud.com (B.A.); peter.stenvinkel@ki.se (P.S.); 3 Department of Global Public Health, Karolinska Institutet, 171 77 Stockholm, Sweden; nicola.orsini@ki.se; 4 Department of Endocrinology, Karolinska University Hospital, 171 76 Stockholm, Sweden; 5 Centre for Diabetes, Academic Specialist Centre, 113 65 Stockholm, Sweden

**Keywords:** fructose loading, inflammatory markers, IL-6, MCP-1, IGFBP-1, type 2 diabetes

## Abstract

Inflammation plays a role in development of diabetic complications. The postprandial state has been linked to chronic low grade inflammation. We therefore aimed to investigate the acute effects of fructose loading, with and without a pizza, on metabolic and inflammatory markers in patients with type 2 diabetes (T2D) (*n* = 7) and in healthy subjects (HS) (*n* = 6), age 47–76 years. Drinks consumed were blueberry drink (18 g fructose), Coca-Cola (17.5 g fructose), and fructose drink (35 g fructose). The levels of glucose, insulin, insulin-like growth factor binding protein-1 (IGFBP-1) and inflammatory markers: Interleukin-6 (IL-6), Monocyte chemoattractant protein-1 (MCP-1), Interleukin-18 (IL-18), Intercellular Adhesion Molecule 1 (ICAM-1), vascular cell adhesion molecule 1 (VCAM-1), and bacterial lipopolysaccharides (LPS) were analyzed in blood. The postprandial responses were assessed using Wilcoxon’s matched-pairs test, Friedman’s ANOVA and Mann–Whitney U test. There was no difference in baseline levels of inflammatory markers between the groups. In T2D, MCP-1 decreased following blueberry drink and Coca-Cola (*p* = 0.02), Coca-Cola + pizza and fructose + pizza (*p* = 0.03). In HS, IL-6 increased following blueberry + pizza and fructose + pizza (*p* = 0.03), there was a decrease in MCP-1 following blueberry drink and Coca-Cola (*p* = 0.03), and in ICAM-1 following blueberry + pizza (*p* = 0.03). These results may indicate a role for MCP-1 as a link between postprandial state and diabetes complications, however further mechanistic studies on larger population of patients with T2D are needed for confirmation of these results.

## 1. Introduction

The consumption of sugar, especially refined or processed fructose in the form of sweeteners in soft and fruit drinks, has increased dramatically in the world [1]. The increased fructose intake has been linked to the development of obesity and type 2 diabetes (T2D) [2,3]. Fructose is predominantly absorbed passively from the intestinal lumen. There is a large individual variation in absorption capacity for fructose. Studies have shown that the presence of glucose is the most important factor affecting the absorption of fructose [4,5], and individuals with T2D have a larger capacity to absorb fructose compared to healthy subjects (HS) [6]. After the absorption, fructose is almost entirely metabolized in the liver in humans [7].

Hepatic fructolysis is unrestricted. As a result of that, fructose load can rapidly be converted to hexose-and trios-phosphate pool leading to a large increase in substrate for glucolysis, gluconeogenesis and lipogenesis [8]. Previous studies have demonstrated that fructose consumption increases fasting triglyceride and glucose levels, stimulates deposition of triglycerides as ectopic fat in other organs and leads to hepatic insulin resistance [4,9,10]. In addition, fructose feeding stimulate purine synthesis and uric acid production [4,7,11]. Previous studies indicate the hyperuricemia has detrimental effect by increasing oxidative stress, inflammation and dyslipidemia [11,12,13], and may be a risk factor for increased cardiovascular disease, worsening of renal failure and mortality [14]. Further, positive associations between uric acid and markers of inflammation have been found [11,14,15,16].

Previous studies have linked postprandial state with chronic systemic low grade inflammation in several diseases including T2D, atherosclerosis and non-alcohol fatty liver disease [17]. Both animal and human studies indicate that inflammation may play a role in pathogenesis of T2D and its’ complications [18]. Previous studies indicate that inflammatory mediators interleukin-6 (IL-6) and interleukin-18 (IL-18), monocyte chemoattractant protein-1 (MCP-1), intercellular adhesion molecule-1 (ICAM-1) and vascular cell adhesion molecule-1 (VCAM-1) may be involved in atherosclerotic cardiovascular disease (CVD), diabetic nephropathy and retinopathy [19,20,21].

The gut microbiota is a complex of microorganism, which in addition to acting as an immune barrier for pathogens also provides numerous beneficial effects for the human host. These include involvement energy metabolism, regulation of host immunity and synthesis of vitamins and amino acids. Diet can affect the gut microbial and intestinal permeability [22,23]. The intestinal permeability can be measured by analyzing bacterial lipopolysaccharides (LPS), an element of the cell walls of Gram-negative bacteria, in serum. Animal studies have shown that high fructose or high glucose diet modulate gut microbial, leading to increased intestinal permeability and endotoxemia and inflammation, causing metabolic disturbance [23,24].

Previous intervention studies, exploring responses of fructose intake on the inflammatory markers IL-6, IL-18, MCP-1, ICAM-1 and VCAM-1 in healthy subject (HS) are scarce and results are conflicting [25,26,27,28,29,30,31]. In addition, many of the studies were performed using high doses of fructose not reflecting real life situations. To the best of our knowledge, there are no previous studies exploring the acute effect of fructose reflecting ad libitum consumption of fructose on inflammatory markers mentioned above in patients with T2D.

In the present study, we aimed to examine the acute postprandial effect of fructose, reflecting ad libitum consumption of fructose, using pure fructose drink (35 g fructose), European Coca-Cola (17.5 g fructose) and a blueberry drink (18 g fructose), without and with a pizza, on IL-6, IL-18, MCP-1, ICAM-1 and VCAM-1 levels in T2D and HS.

Insulin-like growth factor binding protein-1 (IGBP-1) is produced mainly in the liver. The production of IGFBP-1 is strongly regulated by insulin. Fasting levels of IGFBP-1 is a marker of insulin sensitivity in the liver and reduced postprandial suppression of IGFBP-1 predict development of abnormal glucose regulation [32,33]. Therefore, IGFBP-1 and insulin were examined. The Coca-Cola and pizza represent a typical Western meal. As pizza is a meal rich in carbohydrate and fat and will result in hyperglycemia and hypertriglyceridemia, we hypothesized a greater negative effect on inflammatory markers following interventions including pizza, among patients with T2D compared to HS. We further hypothesized that a blueberry drink with antioxidant compounds [34], and lower increase in postprandial serum uric acid compared to Coca-Cola and pure fructose [35], would be more favorable on levels of inflammatory markers.

## 2. Materials and Methods

### 2.1. Study Population

Study participants were recruited at the study sites Department of Endocrinology, Diabetes and Metabolism and Department of Renal Medicine at Karolinska University Hospital, or through advertisements. The inclusion criteria were >18 years of age and diabetes diagnose for patients with T2D. Exclusion criteria were previous cardiovascular events, heart failure, kidney disease, liver disease, ongoing inflammatory disease or infection, treatment with uric acid lowering agents and inability to understand provided study information. In this pilot study, 7 patients with T2D (4 female) and 6 HS subjects (3 female) who completed all interventions were included. The subject characteristics for participants are shown in Table 1. The antidiabetic medication were as follows: 2 were on diet treatment only, 2 were on metformin treatment only, 1 was treated with metformin and a DPP4-inhibitor, 1 was treated with metformin and a glimepride and 1 was treated with metformin and injection NPH insulin at evenings. Patients did not take any antidiabetic medications before the interventions. Three of the patients were on lipid lowering medication. Three of the patients were on antihypertension medication. Three of the patient were on antiplatelet medication ((1) because of previous retina thrombosis ten years before the study and (2) as primary prevention). One patient had hypothyroidism and was on levothyroxin treatment. The study protocol was approved by the Swedish Ethical Review Authority in Stockholm and all study participants signed informed consent. Present study’s Clinicaltrials.gov identifier: NCT03157960.

### 2.2. Interventions

Study participants, who were fasting overnight, ingested a blueberry drink, a Coca-Cola and a fructose drink, with or without a pizza slice, resulting in six interventions in each participant. The isocaloric drinks (140 kcal) contained the following sugars: fructose drink; 35 g fructose (20 cL), Coca-Cola; 17.5 g fructose and 17.5 g glucose (33 cL), and the blueberry drink; 18 g fructose, 14 g glucose and 2–3 g sucrose (54 cL). The blueberry drink was squeezed and pasteurized from fresh Swedish blueberries (Saxhyttegubben Blåbär 100% (<0.5 g protein, <0.5 g fat and 10 g carbohydrate/dL), Saxhytte Gård AB, Grythyttan, Sweden). The Coca-Cola^TM^ was sweetened with the disaccharide sucrose (European formula, 35 g sucrose/33 cL). To standardize the protocol the Coca-Cola cans were kept at room temperature for >4 months during which all sucrose spontaneously decomposed into equal amounts of glucose and fructose [36]. The fructose drink was prepared by dissolving 35 g of pure fructose (>99% pure, Sigma Aldrich, St Louis, MO, USA) in tap water. The fructose and glucose content in drinks, and anthocyanins in blueberry drink were analyzed and have been published previously [35].

Drinks were combined with a pizza slice of 425 kcal (Billy’s pan cheese pizza containing 10% pork, 170 g (17 g protein, 15 g fat and 51 g carbohydrate). Macro distribution do not add up to 170 g due to content of water, spices, preservatives, etc., G. Dafgård AB, Källby, Sweden). The caloric distributions of macronutrients were 32% fat, 51% carbohydrates, 17% protein. Study participants consumed drinks or drink + pizza during 15 min, supervised by a study nurse. The interventions took place between February 2012 and January 2014, and the time interval between interventions ranged between 3 weeks to 9 months (median 43 days). The blueberry drink had a shorter expiry date than other drinks. The Coca-Cola needed to be stored for more than 4 months in room temperature in order for sucrose to be decomposed to glucose and fructose. Therefore, most of the participants started the interventions with blueberry drinks. The order of interventions is shown in the Appendix A (Table A1).

### 2.3. Sample Collection and Laboratory Analysis

Blood samples were collected at −15 min (before intake) and up until 120 min following drinks and 240 min following drinks + pizza. Postprandial responses in serum IL-6, MCP-1 and IGFBP-1 were analyzed for drinks at −15 min and 120 min, and at −15 min and 240 min for drink + pizza. IL-18, ICAM-1, VCAM-1 and LPS (only following Coca-Cola + pizza) were analyzed in plasma for drink + pizza only, at −15 min and 240 min. Glucose and insulin were analyzed at −15 min, 30 min, 60 min, 90 min (glucose only), 120 min and at 240 min. HbA1c, triglycerides, creatinine, fructose, uric acid and hsCRP are presented at baseline. Samples that were not analyzed directly were stored at −80 °C.

Serum IL-6 concentrations were measured on Immulite 1000 Automatic Immunoassay Analyzer (Siemens Healthcare AB, Los Angeles, CA, USA), using an assay manufactured for this analyzer. Concentrations of MCP-1 were analyzed by using ELISA kits (R&D Systems Europe, Ltd., Abingdon, United Kingdom). IL-18, ICAM-1 and VCAM-1 concentrations were measured on LX200 (Luminex Coorp., Austin, TX, USA) using Bio-Plex Pro kit from Bio-Rad. IGFBP-1 concentrations were determined by an in-house RIA according to the method by Póvoa et al. [27]. LPS concentrations were measured on plate reader Envision 2103 (PerkinElmer, Waltham, MA, USA) using Hyglos EndoLISA. Insulin was measured using ELISA-kit (DAKO, Agilent Technologies, Santa Clara, CA, USA). Analysis were performed according to instruction manuals. Analysis of fructose, uric acid, glucose, triglycerides, HbA1c, creatinine, and hsCRP, as well as antioxidant content of the blueberry drink have been described previously [35].

### 2.4. Statistical Analyzes

Descriptive statistics were retrieved from first participating intervention, presented as medians (min, max) or as frequencies and tested with Mann–Whitney U test or Fisher’s exact test (for comparison between two groups) as appropriate. Comparison were made using Mann–Whitney U test (for comparison between 2 groups without normal distribution), Wilcoxon test (for comparisons between 2 related measurements without normal distribution), Friedman’s ANOVA test (for comparisons among 3 or more repeated measurements without normal distribution), and a repeated measures ANOVA (for comparison of differences between interventions over time). Using repeated measure ANOVA, the differences between interventions over time was assessed in each group by interaction term (time × intervention). Measure of insulin resistance, HOMA-IR, was calculated as described by Matthew et al. [37]. Estimated glomerular filtration rate (eGFR) was calculated using CKD-EPI equation [38]. One patient with T2D was excluded from repeated measure ANOVA analysis of insulin due to extreme values (78.3 mIU/L) as the value were outlier and would have contributed to extreme skewness. Within each group, the differences in inflammatory markers and IGFBP-1, between drink interventions and drink + pizza interventions, were determined as percent (%) change between the baseline value and endpoint values. Plausible differences in acute postprandial responses in inflammatory markers, LPS and IGFBP-1, between groups, were determined as % change between the base line values and endpoint values. Statistical significance was set at the level of *p* < 0.05, unadjusted for multiple testing. We decided to not correct for multiple testing as this approach may result in chance associations [39]. Statistical analyzes and figures were made using the analytics software Statistica (Version 13, Dell, Round Rock, TX 78664, USA).

## 3. Results

The baseline characteristics of the study population are presented in Table 1. Patients with T2D had higher levels of serum fructose compared to HS, 107 µmol/L and 72 µmol/L (*p* = 0.022). There were no baseline differences between the groups in serum levels of uric acid, IL-6, IL-18, MCP-1 or CAMs. The weight and HbA1c did not change between the first and last visit in any group.

### 3.1. Postprandial Serum Glucose and Insulin Levels after Drink and Drink + Pizza

**Figure 1:**
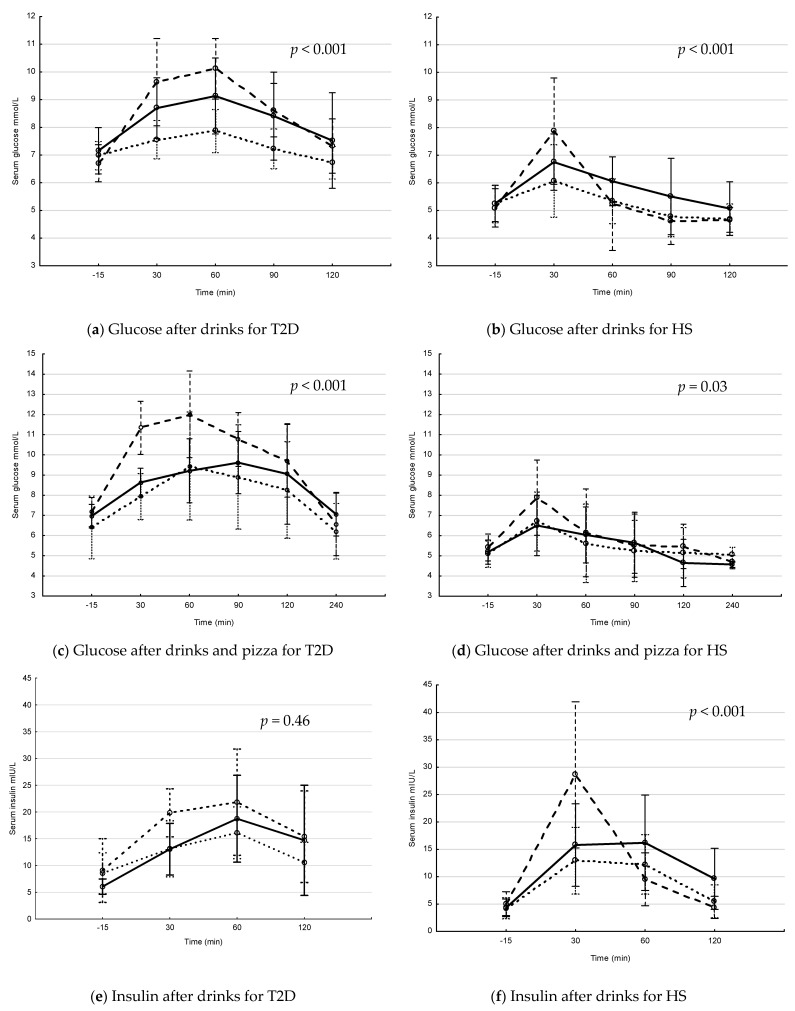

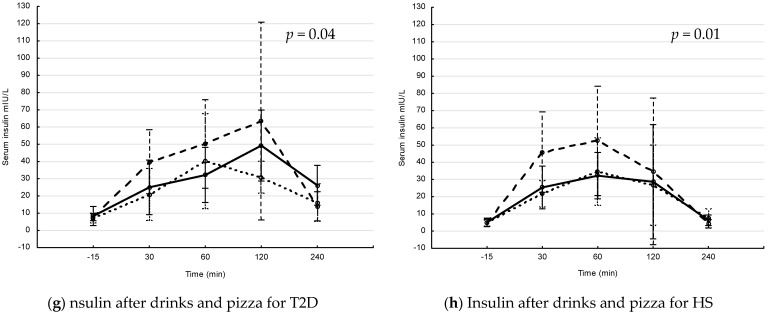
Postprandial glucose and insulin levels in patients with type 2 diabetes (T2D) and healthy subjects (HS) after drinks and drinks + pizza. Mean values with 95% confidence interval. The S-glucose (**a**–**d**) and S-insulin (**e**–**h**) levels in patients with T2D and HS. Solid line = blueberry drink, dashed line = Coca-Cola, dotted line = fructose drink. *p*-values for changes in interventions over time (Repeated measure ANOVA). (**a**) postprandial s-glucose levels after drink interventions in T2D, (**b**) postprandial s-glucose levels after drink interventions in HS, (**c**) postprandial glucose levels after drink + pizza interventions in T2D, (**d**) postprandial s-glucose levels after drink + pizza interventions in HS, (**e**) postprandial s-insulin levels after drink interventions in T2D, (**f**) postprandial s-insulin levels after drink interventions in HS, (**g**) postprandial s-insulin levels after drink + pizza interventions in T2D and (**h**) postprandial s-insulin levels after drink + pizza interventions in HS.

The postprandial levels of serum glucose and insulin after interventions with drink and drink + pizza are shown in Figure 1. In patients with T2D, the postprandial s-glucose levels increased over time, with differences between interventions after drinks (Figure 1a) and drinks + pizza (Figure 1c). The postprandial s-glucose levels were significantly higher after Coca-Cola and Coca-Cola + pizza compared to interventions with fructose and fructose + pizza (*p* = 0.007 and *p* = 0.029, respectively for repeated measure ANOVA). In HS, the postprandial s-glucose levels increased over time, with differences between interventions over time after drinks (Figure 1b) and drinks + pizza (Figure 1d). The postprandial s-glucose levels were significantly higher after intervention with Coca-Cola + pizza compared to fructose + pizza (*p* = 0.026, repeated measure ANOVA). In patients with T2D, s-insulin levels increased over time, with differences between interventions over time after drink + pizza (Figure 1g). In HS, the s-insulin levels increased significantly over time, with differences between the interventions over time after drinks (Figure 1f) and drinks + pizza (Figure 1h).

### 3.2. Postprandial Changes in Serum IL-6 and MCP-1 after Drinks and Drinks + Pizza

**Figure 2:**
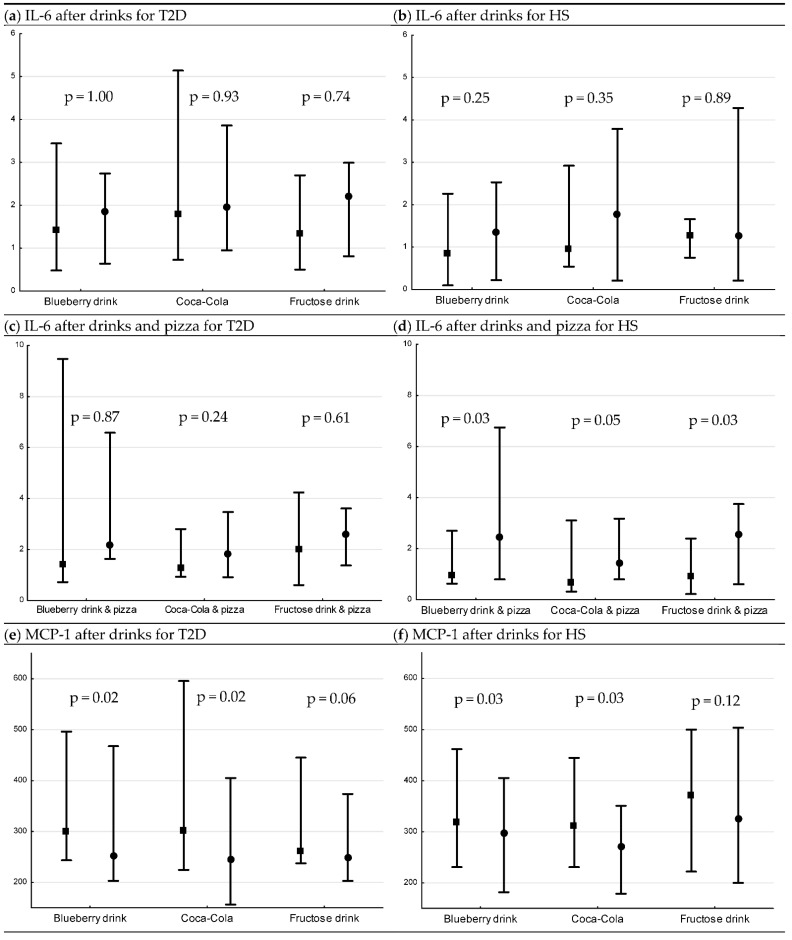

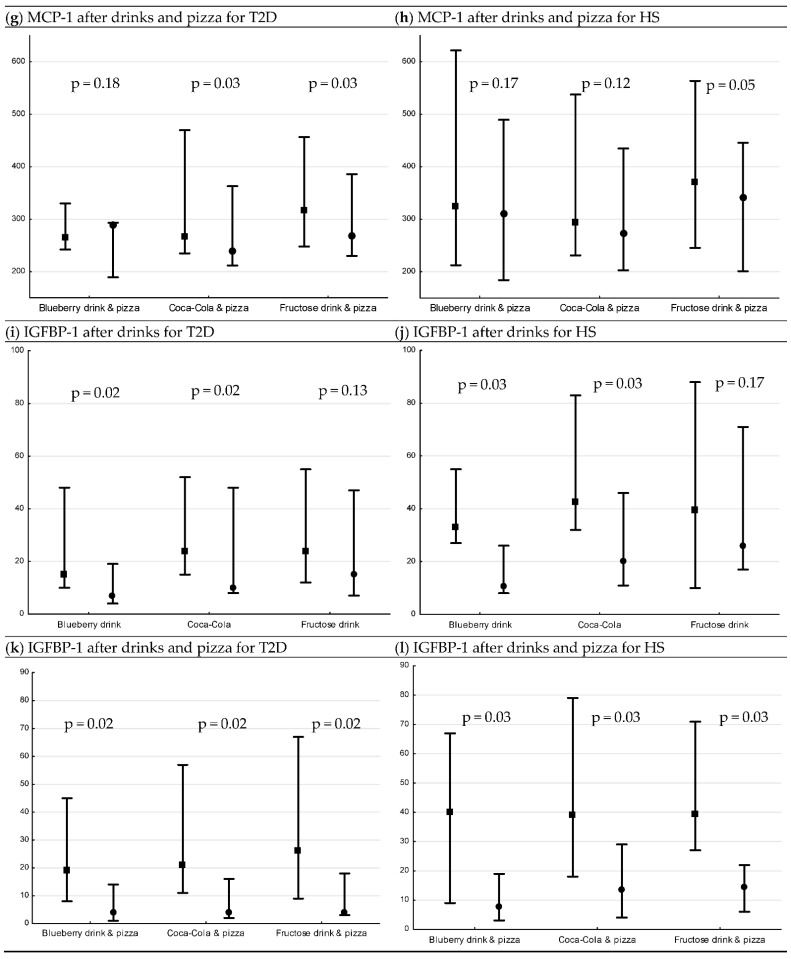
Levels of IL-6, MCP-1 (median pg/mL [min, max]) and IGFBP-1 (median µg/mL [min, max]) 15 min before interventions, and 120 min after drink interventions and 240 min after drink + pizza interventions *. Baseline and endpoint levels of serum IL-6, MCP-1 and IGFBP-1, respectively. *p*-values for Wilcoxon matched pairs test. Square= −15 min, circle = 120 min/240 min, respectively. (**a**) serum IL-6 levels before and after drink interventions in T2D, (**b**) serum IL-6 levels before and after drink interventions in HS, (**c**) serum IL-6 levels before and after drink + pizza interventions in T2D, (**d**) serum IL-6 levels before and after drink + pizza interventions in HS, (**e**) serum MCP-1 levels before and after drink interventions in T2D, (**f**) serum MCP-1 levels before and after drink interventions in HS, (**g**) serum MCP-1 levels before and after drink + pizza interventions in T2D, (**h**) serum MCP-1 levels before and after drink + pizza interventions in HS, (**i**) serum IGFBP-1 levels before and after drink interventions in T2D, (**j**) serum IGFBP-1 levels before and after drink interventions in HS, (**k**) serum IGFBP-1 levels before and after drink + pizza interventions in T2D and (**l**) serum IGFBP-1 levels before and after drink + pizza interventions in HS.

There were no changes in serum levels of IL-6 following drink interventions in any group (T2D; Figure 2a, HS; Figure 2b). The IL-6 levels increased significantly in HS after blueberry + pizza and fructose + pizza (Figure 2d), and there was an increase after Coca-Cola + pizza but the difference did not reach significant levels (*p* = 0.05). In contrast, no changes in postprandial IL-6 was observed in patients with T2D (Figure 2c).

There were no differences in the postprandial percent change in IL-6 for drinks or drinks + pizza, respectively, between or within groups.

A decrease in serum levels of MCP-1 was observed following blueberry drink and Coca-Cola in both patients with T2D and HS (T2D; both *p* = 0.02 (Figure 2e), HS; both *p* = 0.03 (Figure 2f)). When pizza was combined with the drinks, a decrease in postprandial levels of MCP1 was observed following Coca-Cola (*p* = 0.03, Figure 2g) and fructose (*p* = 0.03, Figure 2g) only in patients with T2D. No changes were observed in postprandial levels of MCP-1 in HS (Figure 2h).

For HS, MCP-1 had a greater postprandial percent decrease following Coca-Cola compared to fructose drink (*p* = 0.03, post hoc analysis). There were no other differences observed in postprandial percent change in MCP-1 for drinks or drinks + pizza, respectively, between or within the groups.

### 3.3. Postprandial Changes in Serum IGFBP-1 after Drinks and Drinks + Pizza

There was a decrease in serum levels of IGFBP-1 following blueberry drink and Coca-Cola in both patients with T2D and HS (T2D; both *p* = 0.02 (Figure 2i), HS; both *p* = 0.03 (Figure 2j)). When pizza was combined with the drinks, there was a decrease following all interventions (T2D; all *p* = 0.02 (Figure 2k), HS; all *p* = 0.03 (Figure 2l)). For HS, IGFBP-1 had a greater postprandial decrease following blueberry drink compared to fructose drink (*p* = 0.002, post hoc analysis).

There were no other differences observed in postprandial percent change in IGFBP-1 for drinks or drinks + pizza, respectively, between or within groups.

### 3.4. Postprandial Changes in Plasma ICAM-1, VCAM-1 and IL-18 after Drink + Pizza

**Figure 3:**
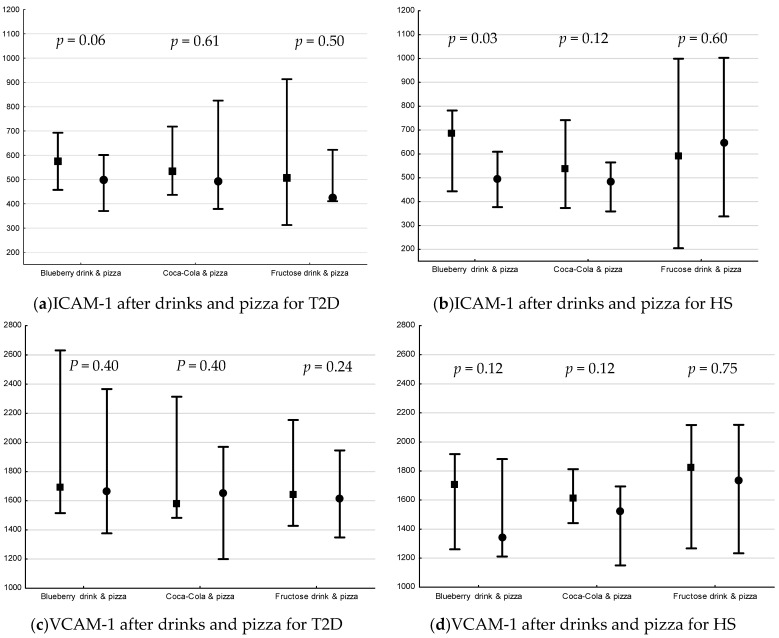

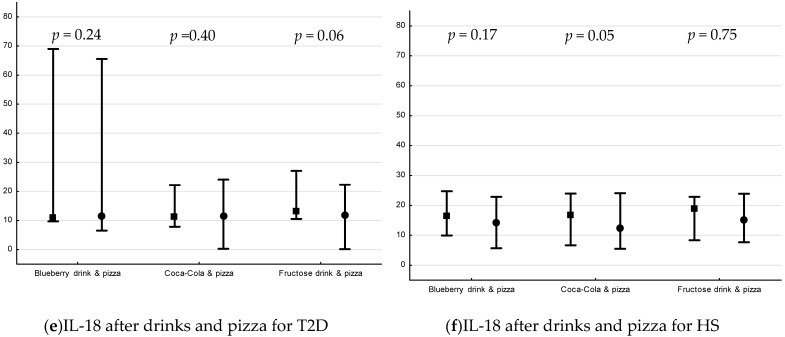
Plasma levels (median pg/mL [min, max]) of ICAM-1, VCAM-1 and IL-18 15 min before interventions and 240 min after drink + pizza interventions *. Baseline and endpoint levels of ICAM-1, VCAM-1 and IL-18, respectively. *p*-values for Wilcoxon matched pairs test. Square= −15 min, circle = 240 min, respectively. (**a**) plasma ICAM-1 levels before and after drink + pizza intervention in T2D, (**b**) plasma ICAM-1 levels before and after drink + pizza interventions in HS, (**c**) plasma VCAM-1 levels before and after drink + pizza in T2D, (**d**) plasma VCAM-1 levels before and after drink + pizza in HS, (**e**) plasma IL-18 levels before and after drink + pizza interventions in T2D, and (**f**) plasma VCAM-1 levels before and after drink + pizza in HS.

The postprandial changes in plasma ICAM-1, VCAM-1 and IL-18 are shown in Figure 3. There was a decrease only in plasma levels of ICAM-1 in HS following blueberry drink + pizza (*p* = 0.03, Figure 3b).

There was no difference in postprandial percent change in plasma IL-18, ICAM-1 or VCAM-1 for drinks + pizza between or within groups.

### 3.5. Postprandial Changes in LPS after Coca-Cola + Pizza

No changes were observed in plasma levels of LPS following intake of Coca-Cola + pizza (HS; *p* = 0.05, T2D; *p* = 0.40), and there was no difference in postprandial percent change of LPS between the groups.

## 4. Discussion

In this present study, there were no differences in basal levels of inflammatory markers (IL-6, Il-18, MCP-1, I-CAM or VCAM-1) between patients with T2D and HS. An acute effect of fructose load, reflecting ad libitum consumption of fructose, was observed on the inflammatory marker MCP-1. When combining fructose-containing drinks with pizza, reflecting ad libitum consumption of fructose and Western diet, an acute decrease in MCP-1 was observed in T2D but not in HS.

In this study, comparing patients with T2D to HS, there were no differences in baseline levels of inflammatory markers, which are in contrast with some previous observations [40,41,42,43]. These findings may partly be explained by a well-controlled diabetes among patients with T2D in present study as the median HbA1c was within acceptable range.

In the present study, levels of serum MCP-1 decreased in both groups following Coca-Cola and blueberry drink. When pizza was combined with drinks, the decrease was observed only in patients with T2D following Coca-Cola and fructose drink (Figure 2). Previous long term studies examining serum MCP-1 following fructose intake in healthy, and overweight and obese subjects show no changes or increase in levels of serum MCP-1, following a four week and a 10 week, respectively, intervention [25,27]. MCP-1 is produced by a variety of cell types, either constitutively or after induction to oxidative stress [44]. Studies have linked MCP-1 to cardiovascular disease and to insulin resistance [44,45,46]. Studies indicate that MCP-1 induces its effect locally by infiltrating to the arterial wall and initiating the atherosclerotic process [45] or inducing local inflammation in adipose tissue resulting in insulin resistance [46]. To our knowledge, there are no other studies investigating the postprandial MCP-1 levels in patients with T2D. The decrease in MCP-1 levels observed in this study may suggest a translocation of MCP-1 in the arterial walls. These results may indicate that serum MCP-1 may have a role for linking postprandial state to diabetes complications.

Our finding of no increase in IL-6 following pure drinks support finding by Mah et al. of postprandial response among HS [31]. Increase in glucose and triglycerides levels in the postprandial state can stimulate inflammation [47], which might explain the increase in IL-6 in HS following intervention including pizza (Figure 2). The delayed glucose response in patients with T2D might explain the absence of an increase in IL-6, and a follow-up of 240 min might thus be too short. Inflammatory responses following carbohydrate rich meal show inconsistent results among HS and T2D [40,41,48,49,50].

The postprandial effect of fructose on levels of ICAM-1, VCAM-1 and IL-18 were in this present study investigated in interventions including pizza. A decrease in ICAM-1 was observed following blueberry drink + pizza in HS only (Figure 3). Nappo et al. observed an increase in ICAM-1 and VCAM-1 only among T2D following a high carbohydrate meal (~73% of energy), which was prevented with addition of vitamins [41]. The amount of carbohydrate in the meal in this study was lower which may explain the differences in the results. There was no response in IL-18 in the present study, which is in line with previous studies (carbohydrates; ~70% [low fiber] and ~76% of energy, respectively) [42,48].

In this study, there were no postprandial changes in serum levels of LPS. This is in contrast to previous studies; however, the differences may be explained by a possible greater effect, i.e., increase, previously observed following a diet high in saturated fatty acids [51]. For IGFBP-1, a marker of hepatic insulin sensitivity, there was no difference between patients with T2D and HS, indicating that patients with T2D in this study had not significant hepatic insulin resistance [32]. The levels of IGFBP-1 decreased significantly after the blueberry and Coca-Cola and after drinks + pizza but not after the fructose drink, which may be due to lower increase in insulin levels after fructose drink only (Figure 1).

Some strengths and limitations should be considered when interpreting the results in this present study. The strengths of the study include the use of three different fructose sources, where the blueberry drink represents the supposedly healthier alternative, and the combination of Coca-Cola and pizza represent a typical Western meal. Further, as the monosaccharides fructose and glucose may be more detrimental than the disaccharide sucrose, the breakdown of sucrose in European Coca-Cola was taken into account to avoid incorrect interpretations [52]. Moreover, to the best of our knowledge, there are no previous studies examining the acute effect of fructose loading on IL-6, MCP-1, ICAM, VCAM-1 and IL-18 among patients with T2D. Among the limitations, it should be acknowledged that the results are based on a small number of participants. As acknowledged previously [35], this may reduce the power in the study and increase the probability of type 2 error, i.e., we fail to reject the null hypothesis. This may have an impact on the possibility of detecting true differences. Due to the choice of using iso-caloric drinks, there was a double amount of fructose in the fructose drink compared to the other drinks, and for ICAM-1, VCAM-1 and IL-18 the pure effect of fructose could not be observed as it was examined in combination with pizza. The longest interval between the first and last visit was 9 months. A long interval may confound the results. However, the weight and HbA1c was measured at each visit. There was no significant change in weight or HbA1c between the first and last visit in any group.

The group of patients with T2D in this study were heterogeneous regarding their age and the use of medications, which may have an impact on the results. However, patients with T2D are a heterogeneous population in general. The patient group in this study had no renal insufficiency or previous history of cardiovascular disease. The HBa1c levels for all patients were below 60 mmol/mol indicating an acceptable metabolic control, implicating that the results observed in this study may not apply to other T2D populations.

In conclusion, in the current study there were no differences in fasting levels of inflammatory markers (IL-6, IL-18, MCP-1, ICAM-1 or VCAM) between patients with T2D and HS. A well-controlled diabetes may explain these results. An acute effect of fructose load was observed in the postprandial levels of IL-6 and ICAM-1 in HS only and in the postprandial levels of serum MCP-1 in both HS and patients with T2D. When combining fructose-containing drinks with pizza, an acute decrease in serum MCP-1 was observed in patients with T2D but not in HS. These results may indicate a role for MCP-1 as a link between postprandial state and diabetes complications, however further mechanistic studies on larger population of patients with T2D are needed for confirmation of these results.

## Figures and Tables

**Table 1:** Characteristics of study population *.

	HS *n* = 6	T2D *n* = 7	*p*-Value
Age, years	59 (47; 71)	67 (58; 76)	0.101
Male/female, *n*	3/3	3/4	1.000
BMI, kg/m^2^	25.5 (23.0; 27.0)	27.9 (26.1; 33.3)	0.008
SBP, mmHg	125 (102; 145)	140 (120; 140)	0.234
DBP, mmHg	78 (69; 87)	80 (70; 80)	0.534
Glucose, mmol/L	5.4 (4.2; 6.0)	6.9 (5.8; 7.7)	0.002
HbA1c, mmol/mol	38 (33; 47)	50 (43; 57)	0.008
Insulin, mIU/L	4.7 (1.6; 6.2)	6.1 (4.3; 78.3)	0.051
HOMA-IR	1.1 (0.3; 1.7)	1.9 (1.4; 23.3)	0.005
Triglycerides, mmol/L	1.0 (0.8; 1.3)	1.1 (0.9; 1.7)	0.366
Creatinine, µmol/L	81 (61; 88)	74 (47; 108)	0.945
eGFR, mL/min/1.73 m^2^	82 (75; 104)	75 (57; 112)	0.445
Fructose, µmol/L	72 (50; 88)	107 (75; 124)	0.022
Uric acid, µmol/L	317 (185; 404)	339 (241; 471)	0.534
hsCRP, mg/L	0.6 (0.4; 1.8)	0.8 (0.5; 3.4)	0.181
MCP-1, pg/mL	319 (231; 462)	300 (243; 445)	0.731
IL-6, pg/mL	0.9 (0.1; 2.3)	1.4 (0.5; 2.7)	0.101
IL-18, pg/mL	16 (10; 25)	12 (10; 33)	0.628
ICAM-1, pg/mL	685 (443; 782)	552 (457; 618)	0.051
VCAM-1, pg/mL	1704 (1261; 1916)	1639 (1472; 2154)	0.836
IGFBP-1	33 (27; 55)	18 (10; 48)	0.073
Diabetes duration, years	-	10 (8; 14)	-

* Values are median (min, max) unless otherwise indicated. Possible differences between the groups tested with Mann–Whitney or Fishers exact test as appropriate. Baseline values of lipopolysaccharides are not presented due to analysis related plate-differences.

**Figure A1:**
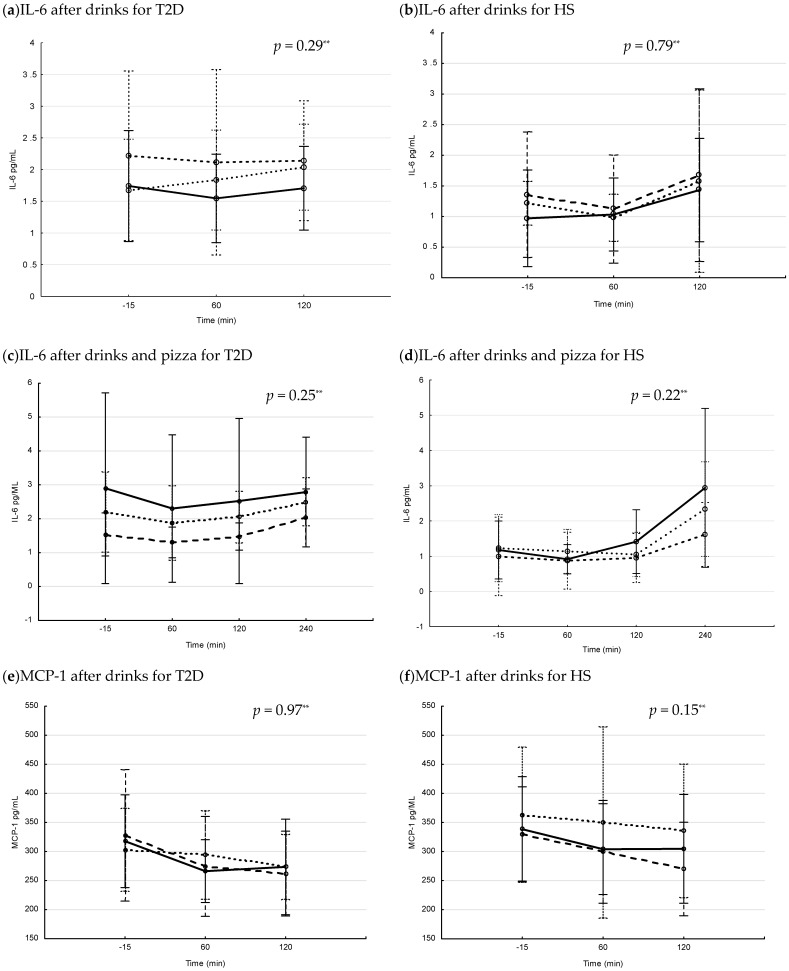

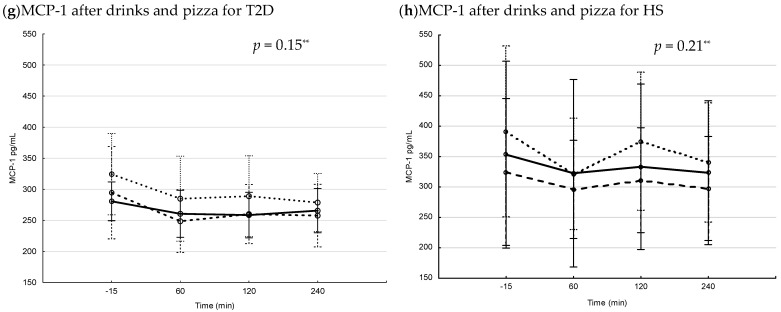
Postprandial serum IL-6 and MCP-1 levels in patients with type 2 diabetes (T2D) and healthy subjects (HS) after drinks and drinks + pizza. Mean values with 95% confidence interval. The S-IL-6 (**a**–**d**) and MCP-1 (**e**–**h**) levels after interventions with drinks and drinks + pizza in subjects with T2D and HS. Solid line = blueberry drink, dashed line = Coca-Cola, dotted line = fructose drink. *p*-values changes in interventions over time (Repeated measure ANOVA). ** = significant differences between interventions (Repeated measure ANOVA, when interaction term for Time × Intervention was found insignificant).

## Data Availability

The data presented in this study are available on request from the corresponding author. The data are not publicly available due to ethical restrictions.

## Appendix A

**Table A1:** Interventions order.

Participant	Visit 1	Visit 2	Visit 3	Visit 4	Visit 5	Visit 6
HS1	BB	BB + P	F	F + P	CC	CC + P
HS2	BB	BB + P	CC + P	CC	F + P	F
HS3	BB	BB + P	F	F + P	CC	CC + P
HS4	BB	BB + P	CC	F	F + P	CC + P
HS5	BB	BB + P	CC	F	CC + P	F + P
HS6	BB	BB + P	CC	CC + P	F	F + P
T2D1	BB	BB + P	CC	CC + P	F	F + P
T2D2	BB	BB + P	CC	F	CC + P	F + P
T2D3	BB	BB + P	CC	F	F + P	CC + P
T2D4	BB	BB + P	CC	F	F + P	CC + P
T2D5	BB	F	F + P	CC	BB + P	CC + P
T2D6	BB	F	CC	F + P	CC + P	BB + P
T2D7	F	BB	CC	F + P	CC + P	BB + P

The order of interventions in participants. HS = Healthy Subject, T2D = Patient with type 2 diabetes, BB = Blueberry drink, F = Fructose drink, CC = Coca Cola, +P = + Pizza.
